# Social Opportunity Rapidly Regulates Expression of CRF and CRF Receptors in the Brain during Social Ascent of a Teleost Fish, *Astatotilapia burtoni*

**DOI:** 10.1371/journal.pone.0096632

**Published:** 2014-05-13

**Authors:** Russ E. Carpenter, Karen P. Maruska, Lisa Becker, Russell D. Fernald

**Affiliations:** Biology Department, Stanford University, Stanford, California, United States of America; University of Tennessee, United States of America

## Abstract

In social animals, hierarchical rank governs food availability, territorial rights and breeding access. Rank order can change rapidly and typically depends on dynamic aggressive interactions. Since the neuromodulator corticotrophin releasing factor (CRF) integrates internal and external cues to regulate the hypothalamic-pituitary adrenal (HPA) axis, we analyzed the CRF system during social encounters related to status. We used a particularly suitable animal model, African cichlid fish, *Astatotilapia burtoni*, whose social status regulates reproduction. When presented with an opportunity to rise in rank, subordinate *A. burtoni* males rapidly change coloration, behavior, and their physiology to support a new role as dominant, reproductively active fish. Although changes in gonadotropin-releasing hormone (GnRH1), the key reproductive molecular actor, have been analyzed during social ascent, little is known about the roles of CRF and the HPA axis during transitions. Experimentally enabling males to ascend in social rank, we measured changes in plasma cortisol and the CRF system in specific brain regions 15 minutes after onset of social ascent. Plasma cortisol levels in ascending fish were lower than subordinate conspecifics, but similar to levels in dominant animals. In the preoptic area (POA), where GnRH1 cells are located, and in the pituitary gland, *CRF* and *CRF_1_* receptor mRNA levels are rapidly down regulated in ascending males compared to subordinates. In the Vc/Vl, a forebrain region where CRF cell bodies are located, mRNA coding for both *CRFR_1_* and *CRFR_2_* receptors is lower in ascending fish compared to stable subordinate conspecifics. The rapid time course of these changes (within minutes) suggests that the CRF system is involved in the physiological changes associated with shifts in social status. Since CRF typically has inhibitory effects on the neuroendocrine reproductive axis in vertebrates, this attenuation of CRF activity may allow rapid activation of the reproductive axis and facilitate the transition to dominance.

## Introduction

Social interactions shape the behavior, physiology and reproductive capacity of many animals, especially those living in social groups characterized by dominance hierarchies. In such species, lower social rank is associated with submissive behavior, reduced reproductive opportunity, and increased hypothalamic-pituitary adrenal (HPA) (hypothalamic-pituitary-interrenal, HPI, axis in fishes) axis activity. The HPA/I axis, which signals via the neuropeptide corticotrophin releasing factor (CRF, also known as corticotrophin releasing hormone, CRH), is well known for its role in the stress response [1]. In addition, this HPA/I axis coordinates behavioral, endocrine, autonomic, immune and reproductive responses to stressors [2]. Subordinate individuals typically have increased circulating levels of plasma cortisol, as well as increased CRF activity in the brain [3], [4]. In most social systems, however, hierarchical rank is dynamic, and opportunities for improved rank can result from environmental change as well as direct aggressive interactions [5], [6], [7]. In the case of the social system of the African cichlid fish *Astatotilapia burtoni*, when a subordinate individual ascends in social rank, rapid and dramatic changes occur in behavior (3–5 minutes), physiology, and neural activity (30 minutes) as the individual assumes its new dominant phenotype (reviewed in [5], [8]). In addition, gonadotropin releasing hormone (GnRH1) neurons in the hypothalamus, which are at the apex of the hypothalamic-pituitary-gonadal (HPG) reproductive axis are activated [9]. The timing and downstream effects of social ascent on reproductive physiology have been well documented in *A. burtoni* [10]–[13], but the role of CRF during this status transition has not been examined.

The CRF system is well known for its role in the stress response, and can lead to reduced GnRH1 secretion and reduced reproductive viability [14]–[16]. We hypothesized that central CRF signaling would be greater in low-ranking males, but would decrease as these subordinate males rise in rank to dominant status. To test this hypothesis, we asked how quickly, how much, and in what direction CRF and CRF receptor levels change in the brain during social ascent, particularly in brain regions known to influence social interactions and the HPG axis. CRF is known to initiate release of pituitary adrenocorticotropin (ACTH), which in turn stimulates glucocorticoid secretion [17], and CRF administered centrally in the brain mimics many of the behavioral and autonomic aspects of the stress response (reviewed in [1], [18]). The central CRF and HPA/I systems are highly conserved across vertebrate taxa, and hence likely also involved in regulating social transitions in *A. burtoni*.

Other neuromodulatory systems such as the monoamines (catecholamines and indoleamines) are also well known for their role in influencing a range of behaviors from reward to aggression. Serotonergic signaling, for instance, influences aggressive social behavior and reproductive responsiveness across vertebrate taxa [19], [20]. Since the CRF and serotonergic systems are reciprocally connected, both can influence social and stress-related behavior. For example, in salmonids, CRF and serotonin interact to potentiate locomotion, increase anxiety-like behaviors and decrease latency to social aggression [21]–[23]. In other species, there is some evidence that CRF treatment can lead to a decrease in behaviors associated with reproduction as seen in wild-caught female sparrows, and rodents [24], [25]. Thus, reciprocal interactions between central CRF and serotonergic systems are involved in modulating reproductive and social behaviors in many vertebrates, and make likely candidates for neural regulation of reproductive capacity.

The CRF system plays an important role in species that exist in a dynamic population, governed through social aggression with concomitant stress. In *A. burtoni*, the ability to rise rapidly in social rank produces not only quick behavioral change, but also an increased reproductive capacity. This may act at a neural level as suggested in rodents where there is evidence of CRF-immunoreactive axons contacting the dendrites of GnRH-immunopositive neurons in the mPOA (medial preoptic area), and CRF receptor expression on GnRH1 neurons [26], [27], suggesting that these two systems may be interconnected. CRF release in the POA, therefore, may directly influence reproductive ability. Since the default social status in *A. burtoni* is dominance [28], subordinate males must have neural and physiological mechanisms that specifically inhibit or suppress reproductive and dominance behaviors. Thus CRF is an obvious candidate for regulating GnRH1 and HPG activity during this rise in social rank.

Here we analyzed changes in the CRF signaling system during social ascent from subordinate to dominant status in brain regions that are associated with CRF, GnRH1 and serotonin production and activity. We measured CRF system mRNA levels 15 minutes after perception of a social opportunity because previous studies showed that 30 minutes after ascending, male *A. burtoni* already exhibit increased *GnRH1* mRNA and increases in circulating sex-steroids [13], [29]. We asked whether physiological and neural changes might occur earlier, possibly gating *GnRH1* mRNA and thus HPG activity. We found that mRNA of *CRF* and its receptors change within 15 minutes of social ascent in the pituitary and specific regions of the brain. This rapid response may disinhibit GnRH1 neurons, and/or engage other neural systems to translate social information and opportunity into reproductive competence.

## Materials and Methods

### Animals

Fish used in these experiments were laboratory-bred cichlid fish, *A. burtoni*, derived from wild-caught stock collected in Lake Tanganyika, Africa [28], [30]. They were maintained in aquaria with gravel-covered bottoms and halved terra-cotta flower pots to serve as shelters, and held in environmental conditions that closely mimic their natural habitat (28°C; pH 8.0; 12 hour light: 12 hour dark cycle with full spectrum illumination and constant aeration). Fish were fed daily (AM), and received a diet of cichlid pellets and flakes (AquaDine, Healdsburg, CA, USA). All experimental procedures were approved by the Stanford Administrative Panel for Laboratory Animal Care.

### Social Manipulation

Subordinate males were given the opportunity to ascend in social rank (ascending males) [11], and compared to stable subordinate and dominant control individuals. Briefly, to generate socially and reproductively suppressed individuals prior to social ascent, larger, dominant males socially suppressed smaller experimental subjects in community tanks [31]. Specifically, dominant subject males (standard length, SL: 60.8 2.9 mm; body mass, BM: 5.78±0.99 g) from community tanks were placed into aquaria that contained larger, territorial resident suppressor males (3–4; ∼7.5–9.0 cm SL) and females (4–5; ∼5.0–6.0 cm SL) for 4–5 weeks before experimentation. This ensured complete suppression of the entire reproductive axis in the previously dominant test fish [31]. Visual confirmation of social subordination by focal observations of each fish revealed subordinate behaviors (e.g., fleeing from resident males) and submissive coloration as previously described [9], [11]. Following the suppression period, subjects were moved to the central compartment of a 3-chambered test aquarium that contained one larger resident dominant male (∼7.5–9.0 cm SL) and 3 females (∼5.0–6.5 cm SL). Flanking the central compartment were community tanks that included dominant, subordinate and female fish, separated from the central compartment by clear acrylic barriers so that fish could interact visually but not physically. All dominant males in the adjacent compartments were smaller than the suppressed subject male to assure his ascent when presented with a social opportunity.

Subject males remained in the experimental tank for 2 days, and behavioral observations confirmed that they remained suppressed by the resident male (e.g., subject male performed few to no reproductive or territorial behaviors, and would flee from the resident male when challenged). On the day of ascension (test day), 1 hour before the lights came on, the resident suppressor male was removed from the tank with a net by an experimenter wearing infrared night vision goggles (Bushnell night vision, Model 26–1020). This technique minimized disturbance in the tank, and ensured that the visual and physical absence of the resident suppressor male was observed at light onset for all test subjects.

Stable dominant and subordinate males served as control comparisons to ascending males. Stable subordinate males (SL 61.7±3.7 mm; BM 6.0±1.1 g) were suppressed in community tanks and moved to experimental tanks as described above. On the test day, the net was dipped into the tank prior to light onset to control for disturbance, but the dominant resident was not removed, which kept the existing social hierarchy in place. Stable dominant males (SL 61.2±3.4 mm; BM 6.4±1.1 g) were defined as dominant males that were placed in community tanks with smaller males and females, and allowed to retain their dominant status for 4–5 weeks. When they were moved to the experimental tanks, the central compartment contained 3 females but no larger resident male, and subjects maintained their dominant status. On the test day, a net was dipped into the tank, as described above, but no fish was removed.

### Timing of sampling

All fish were observed by an experimenter (KPM) on test day concurrent with light onset to quantify behaviors and determine time of social ascent. Latency to ascent was calculated as the time between light onset and the time when an individual performed dominance behaviors at a rate of 3 behaviors min^−1^, as described previously [11]. All test fish were sampled 15 minutes after the behavioral ascent threshold was reached. Stable dominant males were also sampled after they performed 3 behaviors min^−1^, while stable subordinate males were sampled at times after light onset that matched the sacrifice times of ascending males.

### Tissue processing

Following observation on test day, all stable dominant, subordinate and ascending males were netted from their tank, briefly anesthetized in ice cold tank water, measured for standard length (±1 mm), weighed (±0.001 g), and blood samples were collected by caudal severance into 100 µl capillary tubes. Blood samples were centrifuged for 10 min (8,000 rpm) and plasma was removed and stored at −80°C until assayed. Pituitaries were collected into 1.5 ml Eppendorf tubes, rapidly frozen on dry ice, and held at −80°C until processing. Whole brains were removed, rapidly frozen on dry ice in mounting media (Neg50, Thermo Scientific) and stored at −80°C until processing. Testes were removed from each fish, weighed, and the gonadosomatic index calculated [GSI =  (gonad mass/body mass)*100].

### Plasma cortisol assay

Plasma cortisol was measured using a commercially available enzyme immuno-assay (Cortisol Express EIA, Cayman Chemical, Inc.) as previously described and validated for this species [32]. Briefly, a 5 µl sample of plasma from each subject was diluted in EIA assay buffer (1∶50–1∶100) and then manufacturer protocols were strictly followed. Plates were read at 405 nm using a microplate reader (UV_max_ Microplate Reader, Molecular Devices), and steroid concentrations determined based on standard curves. All samples were assayed in duplicate and mean coefficients of variation (CV) were 13.9% (intra-assay) and 18.6% (inter-assay).

### Brain microdissection and quantitative reverse transcription-PCR (qRT-PCR)

**Figure 1:**
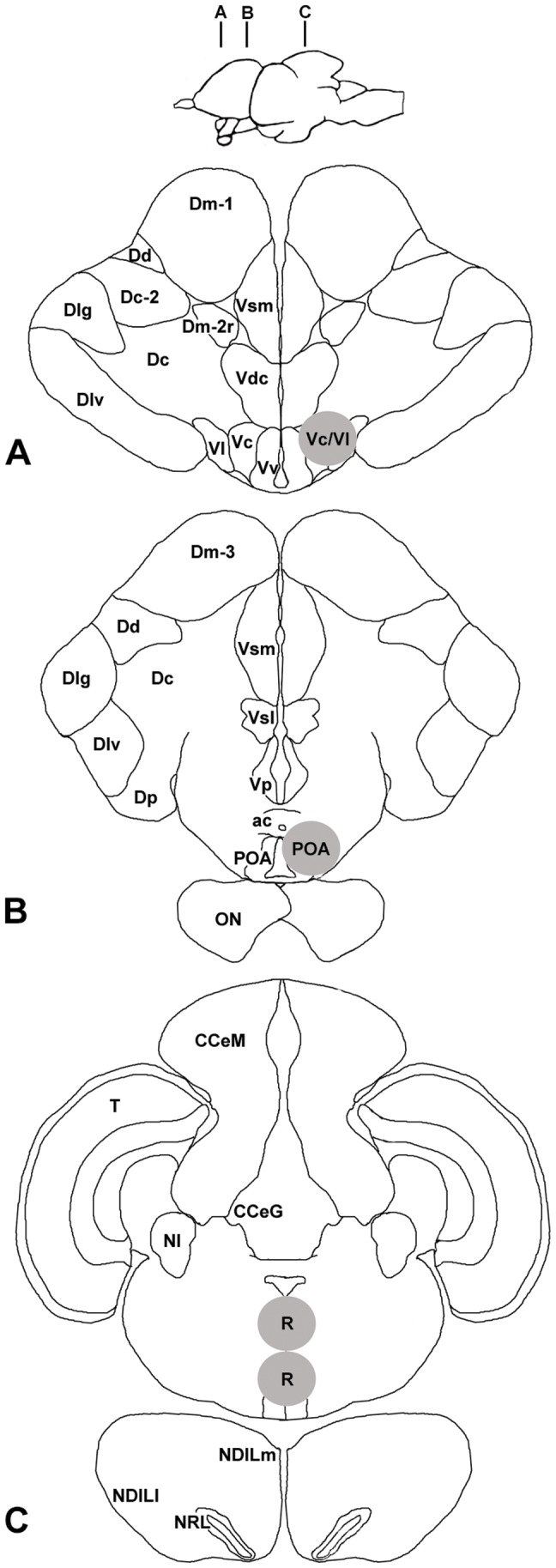
Locations of microdissected brain regions used for qRT-PCR mRNA quantification in the African cichlid *Astatotilapia burtoni*. Top: Schematic sagittal view of the *A. burtoni* brain (rostral facing left) to indicate the approximate locations of coronal sections shown in A–C. **A**) Coronal section through the forebrain, indicating the sampling location for the central and lateral nucleus of the ventral telencephalon (Vc/Vl; grey circle). **B**) Coronal section through the forebrain showing the site of microdissection of the anterior preoptic area (POA; grey circle). **C**) Coronal section through the hindbrain indicating the site for microdissection of the raphè nucleus (R; grey circle). All coronal sections were cut at a thickness of 300 micrometers (µm). *Abbreviations*: ac, anterior commissure; CCeG, Granule cell layer of the corpus cerebelli; CCeM, Molecular layer of the corpus cerebelli; Dc, Central part of the dorsal telencephalon; Dc-2, Subdivision 2 of Dc; Dd, Dorsal nucleus of the dorsal telencephalon; Dlg, Granular division of the lateral part of the dorsal telencephalon; Dlv, Ventral division of the lateral part of the dorsal telencephalon; Dm-1, Subdivision 1 of the medial zone of the dorsal telencephalon; Dm-3; Subdivision 3 of Dm; Dm-2r, Rostral part of Dm-2; Dp, Posterior part of the dorsal telencephalon; NDILl, Lateral part of the diffuse nucleus of the inferior lobe; NDILm, Medial part of the diffuse nucleus of the inferior lobe; NI, Isthmus nucleus; NRL, Nucleus of the lateral recess; ON, Optic nerve; POA, preoptic area; R, Raphé nucleus; T, Tectum; Vc, central nucleus of the ventral telencephalon; Vdc, Caudal part of Vd; Vl, Lateral nucleus of the ventral telencephalon; Vp, Postcommissural nucleus of the ventral telencephalon; Vsm, Medial part of Vs; Vsl, Lateral part of Vs; Vv, Ventral nucleus of the ventral telencephalon.

Fresh frozen whole brains were sectioned coronally at 300 µm in series on a cryostat (Microm HM 550) and briefly thaw mounted onto charged glass microscope slides (Superfrost Plus, VWR). To identify and microdissect specific brain regions, slides were placed on a frozen stage (BFS-30MP, Physitemp) and viewed under a dissection microscope. Tissue was collected with a modified 23G needle (internal diam. 390 µm) attached to a syringe during a single session by the same experimenter (KPM) to reduce sampling variability. To reduce cross-contamination and prevent RNA degradation, the needle was completely cleaned sequentially with RNase-away (Invitrogen, Inc.), ethanol, and RNase-free water between each sample. Brain atlases from *A. burtoni* [33]–[35] and other fish species [36]–[38] were used to target the following brain regions: central nucleus of the ventral telencephalon (Vc/Vl); preoptic area (POA); raphé nucleus (R) (**Fig. 1**). These brain regions were chosen based on their role in CRF production, serotonin production, and HPG axis activity. The Vc has been shown to contain CRF cell bodies in a related cichlid, Tilapia (*Oreochromis mossambicus*) [39], and preliminary results from our lab indicate that *A. burtoni* express CRF cell bodies in this area as well (data not shown). Due to limitations of the microdissection technique, the Vc samples also included the adjacent lateral nucleus of the ventral telencephalon (Vl), and possibly a very small portion of the ventral nucleus of the ventral telencephalon (Vv). The POA contains the only group of GnRH1 neurons in the brain of teleosts, which is the population that directly projects to the pituitary gland to regulate HPG axis activity [40], [41]. We attempted to collect the entire POA as previously described [13], but samples likely did not contain the caudally positioned gigantocellular nucleus. The raphé nucleus was selected because it is the major site of serotonergic production in the brain, is known to modulate stress and aggression [42], [43], and contains CRF receptors [44]–[46]. We sampled both the dorsal and median divisions of the raphe nucleus. The pituitary gland was also examined because it receives direct innervation from the GnRH1 and CRF neurons, and is a common component in the HPG and HPA/I axes [40], [47]. The amount of tissue sampled from each region was standardized across all individuals, collected directly into lysis buffer (RNeasy Micro Plus kit, Qiagen), frozen on dry ice, and stored at −80°C until RNA isolation.

Total RNA was extracted from homogenized pituitaries and microdissected brain tissue using Qiagen RNeasy Plus Micro Kits that included a genomic DNA elimination step (Qiagen, Valencia, CA), and was used to make cDNA by iScript reverse transcription (Bio-Rad Laboratories, Hercules, CA). cDNA was diluted two-fold in nuclease-free water for use in qRT-PCR. Primer sets for all genes were commercially synthesized (Invitrogen) and identical to those used previously for *A. burtoni*: *CRF*, *CRF_1_*, *CRF_2_*, CRF binding protein (*CRFbp*) [48]; *g3pdh*, *18s rRNA* [49]; *LHβ*, *FSHβ* [29], [50]; and *GnRH1* [51], [52]. Each primer pair produced a single melting curve peak in the presence of cDNA template, and showed no or late amplification when water was used as a template in the reaction mix.

Following cDNA synthesis, qRT-PCR was performed in 20-µl duplicate reactions using a Bio-Rad Real-Time PCR system (CFX96) with Ssofast EvaGreen Supermix (Bio-Rad Laboratories, Hercules, CA), 0.25 µM of each primer, and 2.5 ng/µl cDNA. Fluorescence data were processed using Real-time PCR Miner software [49] to calculate threshold cycle number (CT) and efficiency of amplification for each sample. To normalize concentrations, the relative amount of mRNA for each gene of interest, within an individual and within a region, were normalized to the geometric mean of two reference genes, *g3pdh* and *18s rRNA*. Normalization to more than one reference gene can provide a more accurate quantification of mRNA levels for a gene of interest [53], [54]. Values for *g3pdh* and *18s rRNA* were analyzed independently within each region, across groups, via one-way analysis of variance (ANOVA) to verify there was no significant variation among the three test groups being compared. In only one region, the Vc/Vl, *18s rRNA* values differed significantly among groups, so for this brain region, gene of interest data were normalized only to *g3pdh* mRNA. In all other regions (POA, raphé, pituitary) there was no variation difference in *18s rRNA* or *g3pdh* levels among groups, and gene of interest data were normalized to the geometric mean of *g3pdh* and *18s rRNA*.

### Statistical analyses

Data sets that were normally distributed with equal variances (Bartlett's test) were analyzed with one-way ANOVA with *post hoc* Student-Newman-Keuls tests for multiple comparisons. Data that did not meet the assumptions for parametric statistics were compared using the Kruskal-Wallis test with *post hoc* Dunn's tests. For consistency, all data are plotted here as mean ± SEM with the appropriate test values reported in the text. Pearson correlation was used to test for correlations between plasma cortisol levels and GSI. All statistical comparisons were performed within GraphPad Prism 5 (GraphPad Software, Inc., La Jolla, CA).

## Results

### GSI and plasma cortisol levels

**Figure 2:**
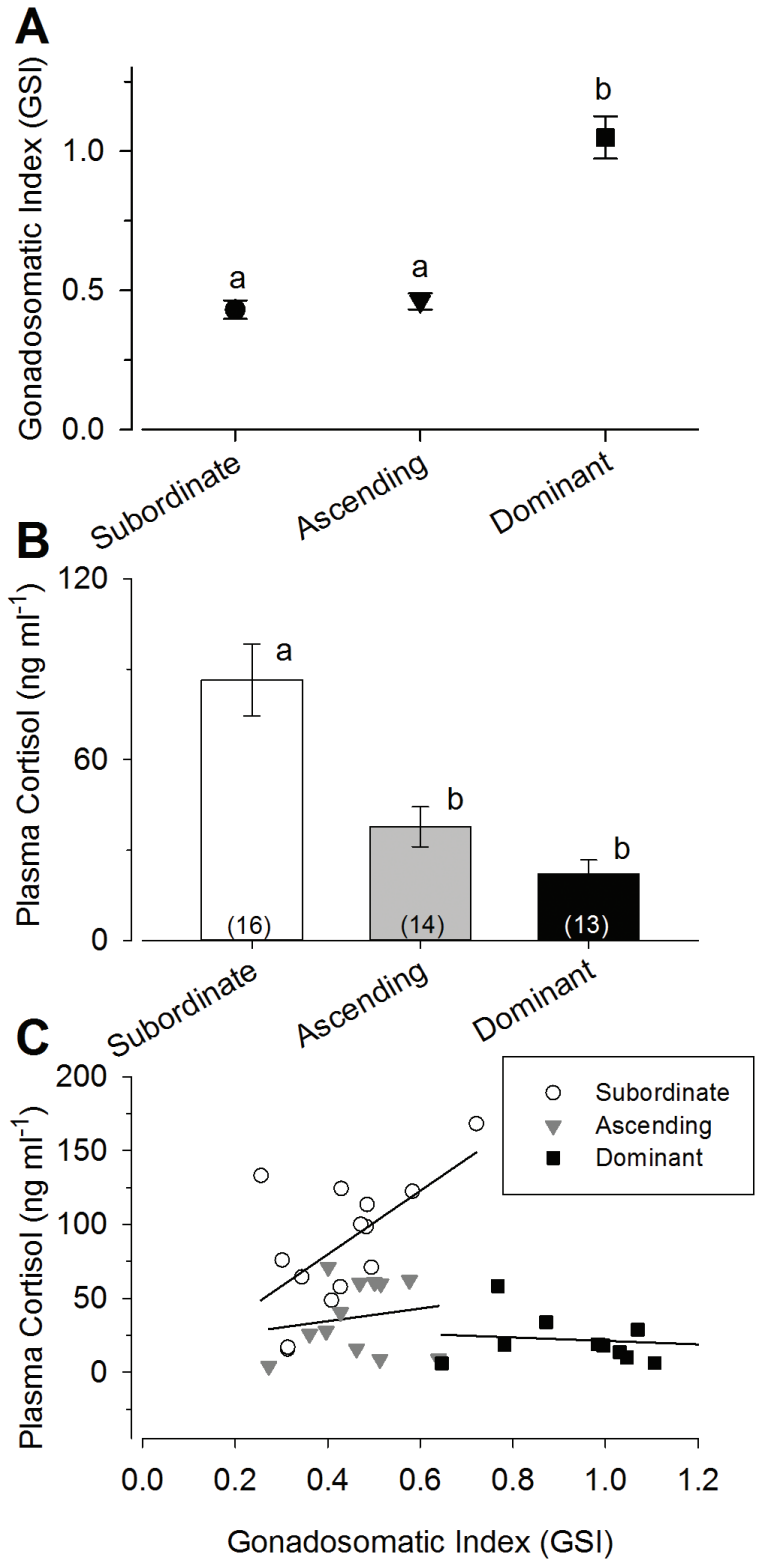
Gonadosomatic index and plasma cortisol levels differed among subordinate, ascending, and dominant male social groups. **A**) Dominant males (*N* = 13) had higher gonadosomatic index values (GSI) compared to both subordinate (*N* = 16) and ascending (N = 14) males. **B**) Plasma cortisol levels were higher in subordinate males compared to both dominant males and males ascending in status 15 minutes after presentation of a social opportunity. Data are plotted as mean ± SEM and sample sizes are indicated in parentheses. Different letters indicate significant differences at *P*<0.05. **C**) Plasma cortisol levels were positively correlated with GSI in subordinate males (open circles), but not in ascending (grey inverted triangles) or dominant (closed squares) males.

The gonadosomatic index, an indication of gonad size relative to body size, was significantly higher in dominant males compared to subordinate and ascending males [ANOVA, F_(2,40)_ = 26.29, P<0.0001; Student-Newman-Keuls, P<0.05], but subordinate and ascending males did not differ from one another, which is not surprising given the short time frame of measurement (**Fig. 2A**).

Circulating cortisol levels were significantly higher in subordinate males compared to both ascending and dominant males, which did not differ from each other [ANOVA, F_2,40_ = 17.41, P<0.0001; Student-Newman-Keuls, P<0.05] (**Fig. 2B**). Plasma cortisol levels were negatively correlated with GSI when all groups were measured together [r = −0.37, 41 d.f., P = 0.022]. When analyzed separately, the subordinate males exhibited a significant positive correlation between plasma cortisol and GSI [r = 0.60, 12 d.f., P = 0.022] (**Fig. 2C**), but no such correlation was found in ascending or dominant males.

### CRF family mRNA levels in the Vc/Vl

**Figure 3:**
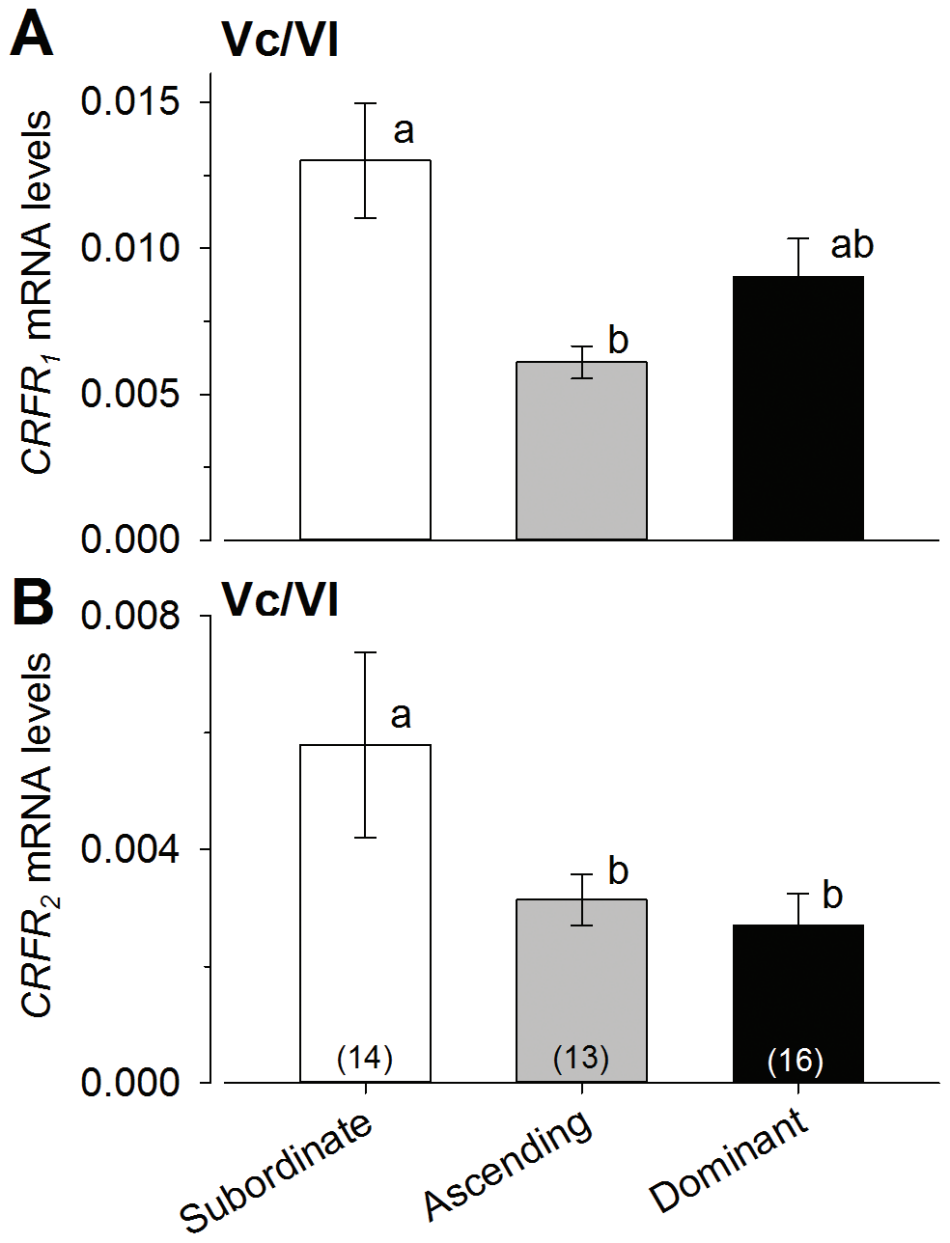
Levels of *CRFR_1_* and *CRFR_2_* mRNA in the central and lateral nuclei of the ventral telencephalon (Vc/Vl) differed among male social groups. **A**) *CRFR_1_* mRNA levels were lower in ascending males at 15 minutes after social opportunity, compared to subordinate males, but neither ascending nor subordinate levels differed from dominant males. **B**) Relative mRNA levels of *CRFR_2_* were significantly lower in dominant males and ascending males at 15 minutes after presentation of a social opportunity compared to subordinate males. mRNA levels were normalized to the reference gene *gapdh*. Data are plotted as mean ± SEM and sample sizes are indicated in parentheses. Different letters indicate significant differences at *P*<0.05.

*CRF_1_* receptor levels in Vc/Vl were lower in ascending males compared to both subordinate and dominant males [ANOVA, F_(2,40)_ = 5.612, P<0.0071; Student-Newman-Keuls, P<0.05] (**Fig. 3A**). *CRF_2_* receptor levels were higher in subordinate males compared to ascending and dominant males [ANOVA, F_(2,38)_ = 3.55, P<0.03; Student-Newman-Keuls, P<0.05] (**Fig. 3B**). There was no difference in *CRF* or *CRFbp* mRNA levels in the forebrain Vc/Vl region among the 3 groups of males [ANOVA, P>0.05] (data not shown).

### CRF family and GnRH_1_ mRNA expression in the POA

**Figure 4:**
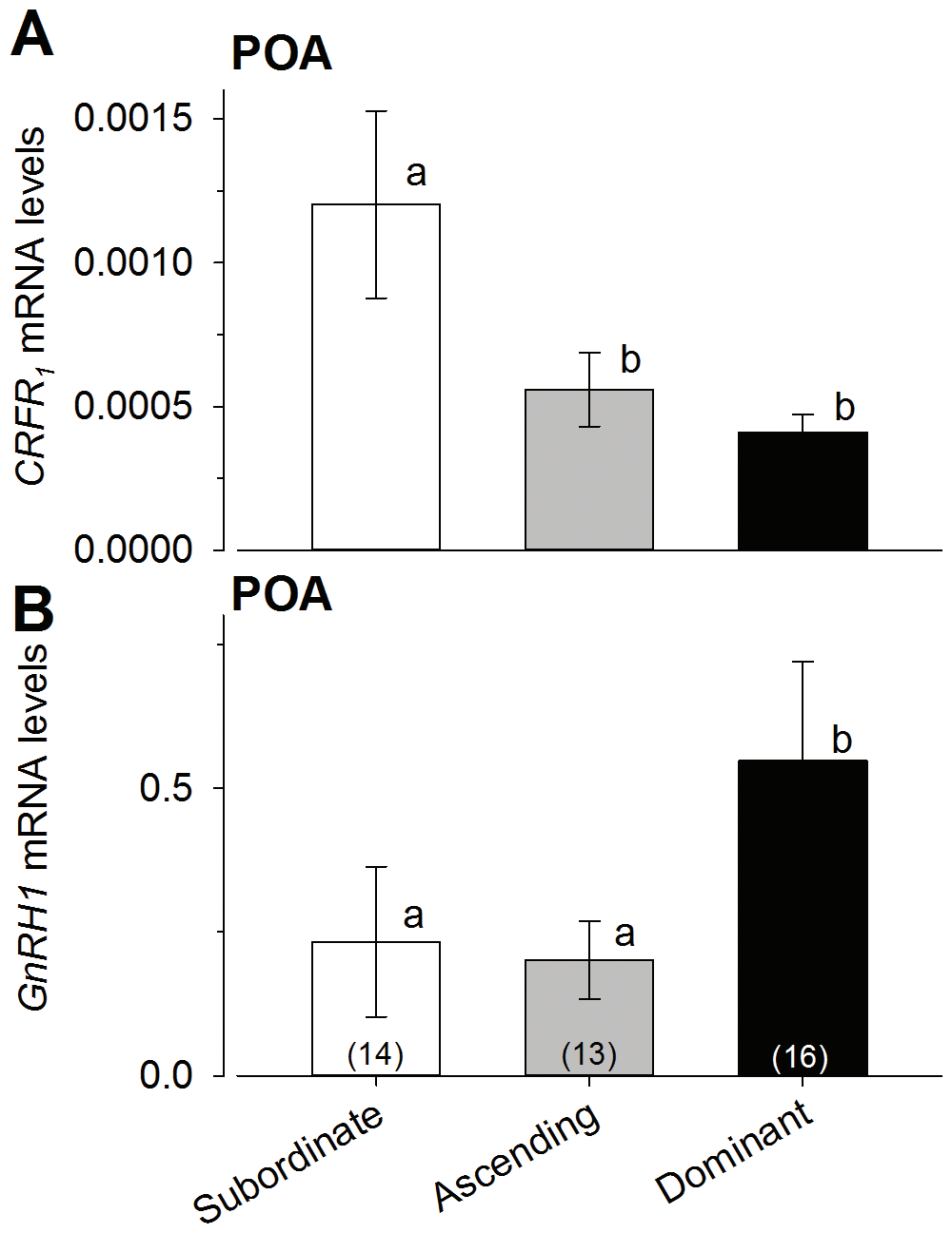
Levels of *CRFR_1_* and *GnRH1* in the preoptic area (POA) differed among male social groups. **A**) *CRFR_1_* mRNA levels in ascending males were similar to dominant males, but both were significantly lower than levels in subordinate males. **B**) Dominant male *A. burtoni* had higher *GnRH1* mRNA levels in the POA compared to both ascending and subordinate male groups. mRNA levels were normalized to the geometric mean of the two reference genes *gapdh and 18s*. Data are plotted as mean ± SEM and sample sizes are indicated in parentheses. Different letters indicate significant differences at *P*<0.05.

Subordinate males expressed significantly higher levels of *CRF_1_* receptor mRNA in the POA compared to ascending and dominant males [ANOVA, F_(2,37)_ = 4.95, P<0.01; Student-Newman-Keuls, P<0.05] (**Fig. 4A**). In contrast, there were no significant differences in *CRF, CRFbp* or *CRF_2_* mRNA levels among groups in the POA [ANOVA, P>0.05] (data not shown).

*GnRH1* mRNA levels were significantly higher in dominant males compared to both subordinate and ascending male groups [ANOVA, F_(2,33)_ = 5.45, P<0.009; Student-Newman-Keuls, P<0.05] (**Fig. 4B**).

### CRF family and gonadotropin mRNA levels in the pituitary

**Figure 5:**
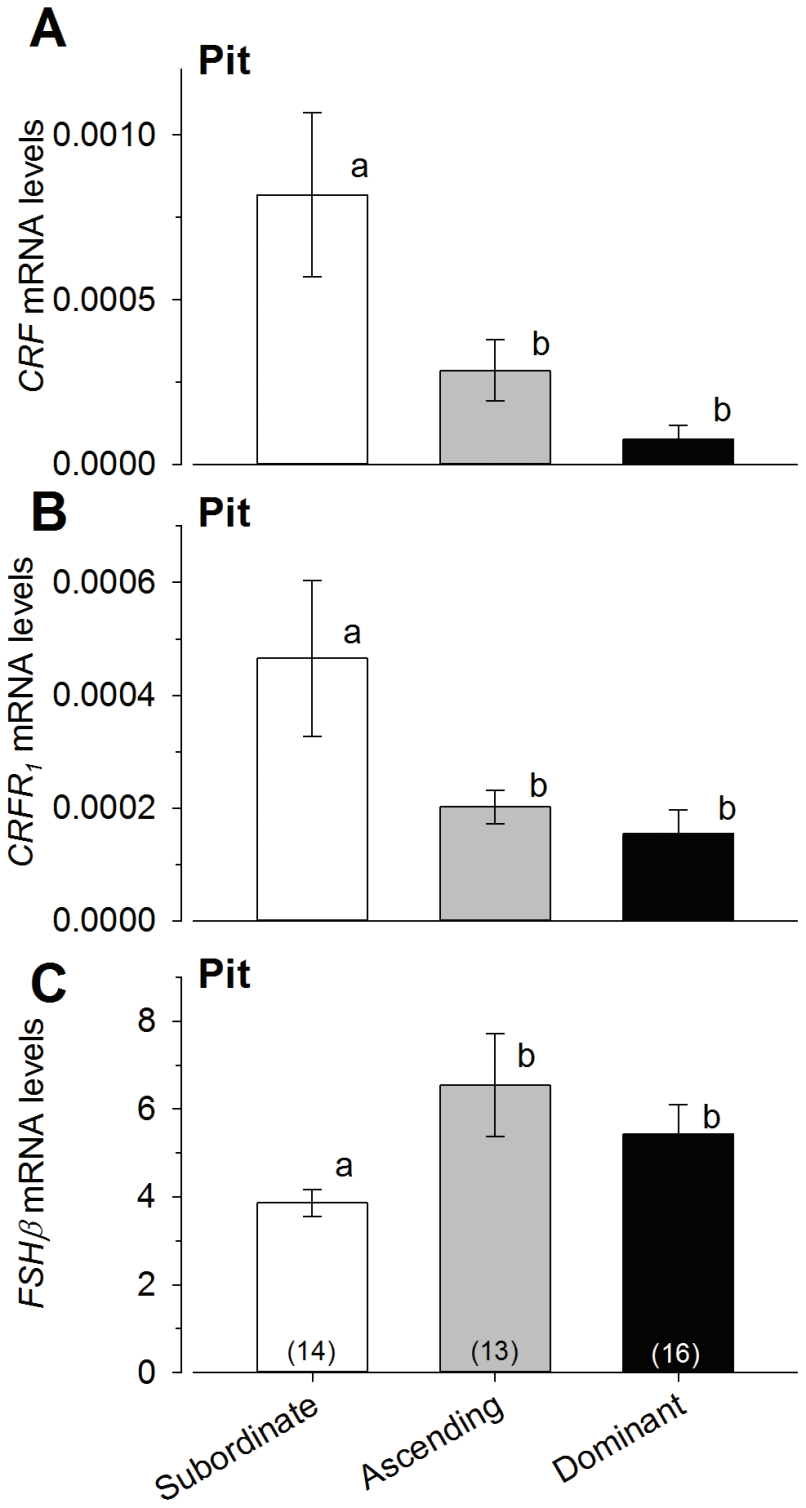
Levels of *CRF*, *CRFR_1_* and *FSHβ* in the pituitary gland differed among male social groups. **A**) Following presentation of a social opportunity, ascending fish showed significantly lower levels of *CRF* mRNA in the pituitary gland compared to subordinate males, but there was no difference between ascending and dominant males. **B**) Similarly, *CRFR_1_* mRNA levels were lower in ascending males at 15 after a social opportunity compared to subordinate males, while ascending and dominant males did not differ. **C**) mRNA levels of the *β*-subunit of the gonadotroph hormone, follicle stimulating hormone (*FSHβ*), were higher in the pituitary of ascending males compared to subordinate males, but similar to levels found in dominant males. mRNA levels were normalized to the geometric mean of the two reference genes *gapdh and 18s*. Data are plotted as mean ± SEM and sample sizes are indicated in parentheses. Different letters indicate significant differences at *P*<0.05.

Levels of *CRF* mRNA in the pituitary were significantly higher in subordinate males compared to both ascending and dominant males [ANOVA, F_(2,38)_ = 7.47, P<0.0018; Student-Newman-Keuls, P<0.05] (**Fig. 5A**), but *CRFbp* mRNA was similar among all three groups [ANOVA, P>0.05] (data not shown). Levels for *CRF_1_* receptor mRNA were significantly higher in subordinate males compared to both ascending and dominant males [ANOVA, F_(2,38)_ = 4049, P<0.01; Student-Newman-Keuls, P<0.05] (**Fig. 5B**). Social groups did not differ in *CRF_2_* mRNA expression [ANOVA, P>0.05] (data not shown).

Levels of *FSHβ* mRNA were significantly elevated in both ascending and dominant males compared to subordinates [ANOVA, F_(2,35)_ = 3.97, P<0.02; Student-Newman-Keuls, P<0.05] (**Fig. 5C**), however, no difference in *LHβ* mRNA was detected among male groups [ANOVA, P>0.05] (data not shown).

### CRF family mRNA levels in the raphé

**Figure 6:**
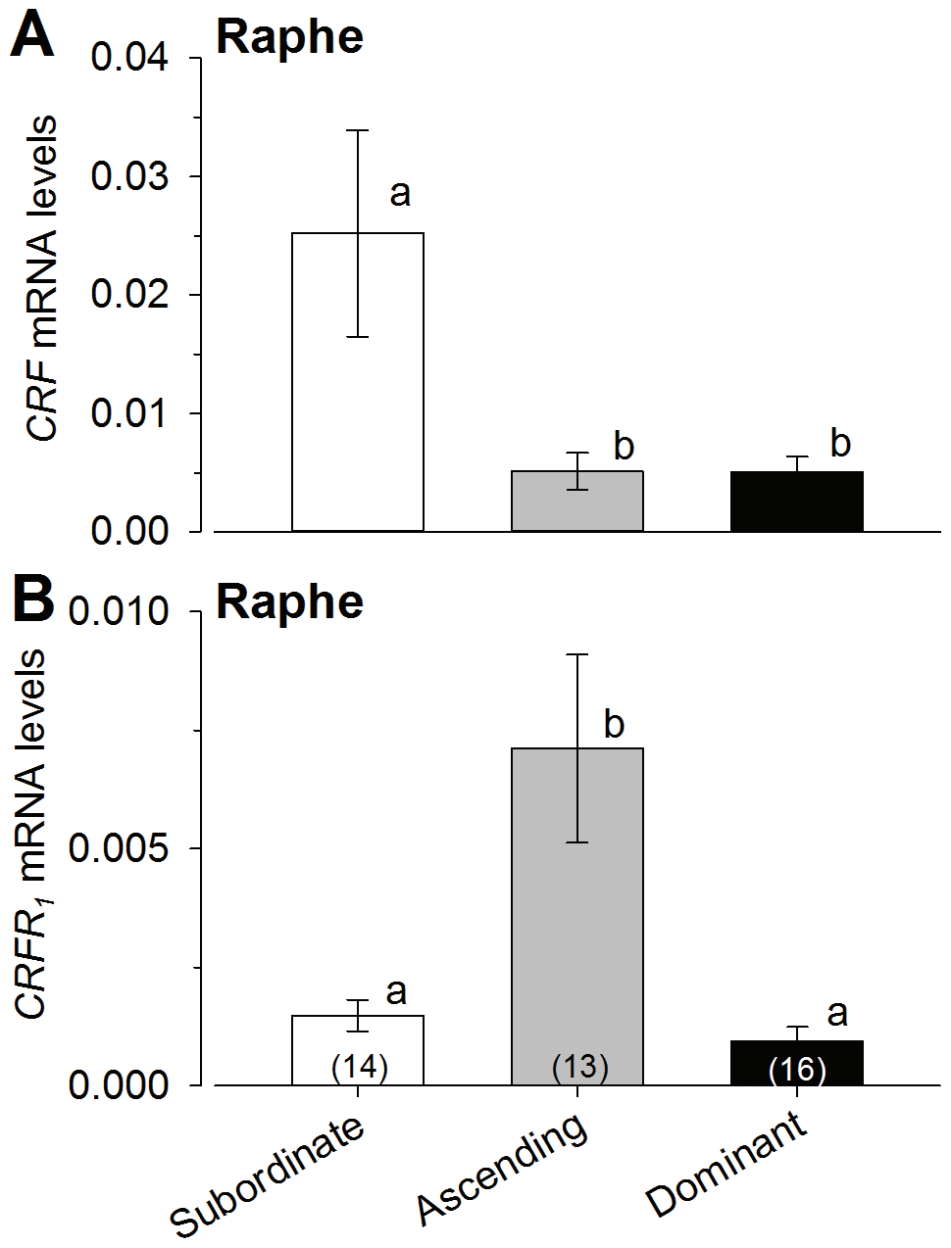
Levels of *CRF* and *CRFR_1_* in the raphé nucleus differed among male social groups. **A**) CRF mRNA levels in the raphé were lower in dominant males and in ascending males at 15 minutes after social opportunity compared to subordinate males. **B**) In contrast, *CRFR_1_* mRNA levels were significantly higher in males ascending in social status compared to both stable subordinate and stable dominant groups. mRNA levels were normalized to the geometric mean of the two reference genes *gapdh and 18s*. Data are plotted as mean ± SEM and sample sizes are indicated in parentheses. Different letters indicate significant differences at *P*<0.05.

*CRF* mRNA was significantly higher in subordinate males compared to both ascending and dominant males in the raphé [ANOVA, F_(2,39)_ = 5.06, P<0.01; Student-Newman-Keuls, P<0.05] (**Fig. 6A**), while there was no difference in *CRFbp* mRNA across groups in this region [ANOVA, P>0.05] (data not shown). Levels of *CRF_2_* receptor mRNA did not vary among groups [ANOVA, P>0.05] (data not shown), however, *CRF_1_* mRNA levels were higher in ascending males compared to both subordinate and dominant males [ANOVA, F_(2,39)_ = 9.44, P<0.0005; Student-Newman-Keuls, P<0.05] (**Fig. 6B**).

## Discussion

**Figure 7:**
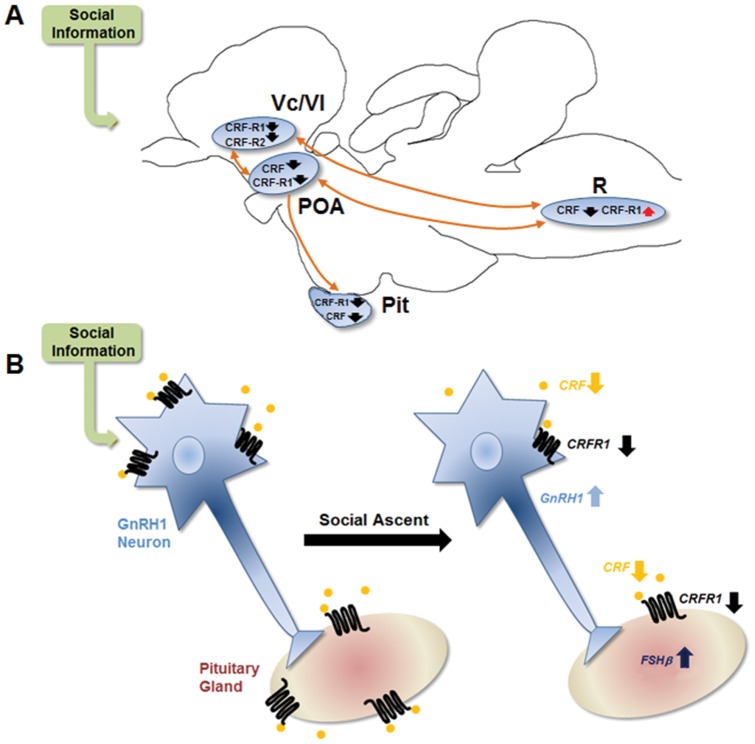
Summary and hypothesized model of the interaction between the stress-related CRF system and reproductive axes during social ascent in male *Astatotilapia burtoni*. Up and down arrows in both panels indicate higher or lower mRNA levels respectively at 15**A**) Schematic summary of measured changes in the CRF system during social ascent. Connections between brain regions are indicated with arrows. **B**) Hypothetical model of the interaction between the CRF-signaling system and GnRH1 neurons in the preoptic area of the brain. At 15 minutes after a subordinate male rises in rank, there are lower mRNA levels of *CRF* and *CRFR_1_* in the GnRH1-containing POA and pituitary gland. This putative reduced CRF sensitivity in the POA may remove the CRF-induced inhibition on GnRH1 neuron and brain-pituitary-gonadal axis activity, leading to increased reproductive capacity associated with dominant status.

Subordinate *A. burtoni*, when presented with an opportunity to rise in social rank, show significant changes in circulating plasma cortisol and brain gene expression of CRF-signaling components within 15 minutes. Plasma cortisol levels in subordinate fish are significantly elevated relative to ascending fish and stable subordinates, suggesting a reduction in HPA/I axis activity when males rise in rank. In both the POA and the pituitary gland, mRNA levels of *CRF* and its receptor *CRFR_1_* are lower in ascending fish relative to stable subordinate conspecifics. In the Vc/Vl forebrain region, homologous in part to mammalian striatal and septal regions, both *CRFR_1_* and *CRFR_2_* mRNA are present at reduced levels in ascending fish compared to non-ascending subordinate controls. Interestingly, in the raphé, the main serotonin producing region in the brain, ascending fish exhibit decreased *CRF* mRNA levels, as well as increased *CRFR_1_* mRNA levels relative to subordinate males (see Fig.7 for summary). Collectively, these results suggest an important role for the CRF-signaling system during social status transition, although they do not distinguish between changes due to, or as a result of, the social rank change.

Social animals that have evolved rank-dependent breeding strategies must have mechanisms that allow them to maintain established hierarchies as well as take advantage of new opportunities to improve their status. When reproductive opportunities are closely tied to status, the ability for a rapid response to social opportunity is important. Our results suggest that disinhibition of neural mechanisms that limit reproductive potency in subordinate animals occurs with rapid social ascent. Previous studies have shown that male *A. burtoni* rising in rank will rapidly (within minutes) begin territorial and reproductive behaviors [9], increase circulating sex steroid levels [8], [13], and elevate pituitary gonadotropins within 30 minutes [11], [12]. These changes indicate that the HPG cascade is rapidly activated, leading to reproductive viability. Here we demonstrate that 15 minutes following a social opportunity, ascending male *A. burtoni* show measureable changes in the HPA/I axis and CRF signaling in the brain. We propose that this may be a mechanism to rapidly facilitate development of reproductive axis activity in ascending individuals. To our knowledge, this is the first study to show that perception of a social opportunity, and response to it, induces such rapid changes in mRNA of the neuropeptide CRF and one of its receptors.

### A role for CRF mediating HPG axis activity

The neuropeptide CRF is well known for its role in activating the HPA/I axis in response to stressors, as well as modulating responses within limbic brain structures [55]. It is also well established that a variety of stressors modulate pulsatile GnRH1 release, and thus, luteinizing hormone (LH) secretion, in a number of species [56]–[58]. Chronic activation of the HPA/I axis results in increased plasma cortisol secretion, known as hypercortisolism, and is associated with disrupted LH secretion in human females [59]. In rats, treatment with glucocorticoids inhibits responsiveness to GnRH without directly influencing GnRH receptor number or activity [60]. Previous work in *A. burtoni* suggests that cortisol may serve as an endogenous signal that relates information about an animal's social environment to its internal reproductive state, and that social instability is tightly linked to elevated cortisol levels [61].

While there is general agreement that HPA axis activity influences GnRH1, FSH and LH activity and release, as noted above, the causal link between plasma corticosteroids and HPG activity is not known. It is likely that CRF plays a more direct role in modulating GnRH1 activity, as populations of CRF neurons and receptors are widely expressed throughout the vertebrate brain [48], [62] and CRF protein levels are elevated following stressful experiences [1]. In trout, restraint stress leads to increased *CRFR_1_* mRNA in the preoptic area (POA), the site of GnRH1 neurons in teleosts [63]. The existence of direct synaptic connections between CRF and GnRH1 neurons in hypothalamic structures has been shown in humans [64] and rats [26] and suggests a possible route for direct functional interaction between these neuropeptide systems. Further, CRF receptors are expressed on subpopulations of POA GnRH1 neurons in mice [27]. In fact, CRF microinfusion into the rat POA inhibits LH release [65] and decreases LH pulse frequency [66], indicating that CRF has the capacity to directly impact the activity of the reproductive axis independent of HPA/I axis activity. Thus, acting centrally within the brain, CRF has the potential to modulate the HPG axis based on external cues and internal physiological state. Since subordinate male *A. burtoni* have elevated levels of *CRF* and *CRFR_1_* mRNA production in the POA (as well as the pituitary and CRF cell body-rich ventral telencephalon) compared to dominant conspecifics, and these expression patterns reverse in such a short time period following social ascent, it seems likely that CRF signaling and HPG axis activity are tightly linked.

### Potential role of CRF inhibition and disinhibition

Here we found that subordinate *A. burtoni* males, subject to inescapable aggressive attacks from dominant, resident conspecifics, show elevated levels of plasma cortisol and *CRF* mRNA in several brain regions. Thus, the reduced *GnRH1* mRNA expression seen in the POA of subordinate *A. burtoni* suggest it is not mediated by HPA/I axis activity alone, and may result from increased CRF activity in the brain. When subordinate males are given an opportunity to ascend in social status, circulating cortisol levels, *CRF* and *CRFR_1_* mRNA expression decreased to a level similar to dominant males in these same brain regions after only 15 minutes. This response suggests that CRF-mediated inhibition in the forebrain, pituitary and hypothalamus may be important for modulating opportunistic behavioral changes and activation of GnRH1 neuronal populations in preparation for a new social role as a dominant, reproductively active male.

Elevated CRF and cortisol signaling in socially subordinate animals may serve as a coping-strategy to maintain reduced reproductive and aggressive activity while living with larger, dominant conspecifics. This strategy would be beneficial to preserve the complex social structure where only approximately 10–30% of the males in a population hold territories and are socially dominant [28]. Yet, rapid disinhibition of this system would be a sufficient and appropriate mechanism to reverse these effects in a short time course when a territory becomes available. It is important to note, however, that in order to disentangle the potential causative nature of this system, further research is needed to determine how the decrease in CRF signaling influences behavioral output and GnRH1 activity. As *CRF* and *CRFR_1_* mRNA rapidly decreased in both the POA and pituitary of ascending fish, as well as *CRFR_1_* in the Vc/Vl forebrain nucleus, a central role for the CRF system seems likely (Fig 7). Further experiments utilizing directed pre-treatment of a CRF agonist *before* an opportunity to socially ascend may reveal the role of this system during social transitions.

### Possible alternative mechanisms

Though the most parsimonious route for CRF modulation of GnRH1 activity would be through direct synaptic contact between these neuron types, it is possible, and even likely, that other direct or indirect mechanisms are also involved. Onset of puberty in mammals is generally characterized by an increase in GnRH1 secretion and concurrent FSH and LH release, which leads to gonadal maturation and appearance of secondary sex characteristics arising from production of sex-steroids [67], [68]. This process results from the activation of the GnRH1 pulse generator, stimulated by functional changes occurring in neuronal and astroglial networks connected to hypothalamic neurons [69]. The primary mode of excitation in the hypothalamus is via glutamate release [70], and GnRH1 neurons receive direct glutamatergic innervation [71]. Treatment with the glutamate agonist n-methyl-d-aspartate (NMDA) in immature animals stimulates GnRH1 secretion in a variety of vertebrates, including goldfish [72], trout [73], rats [74] and monkeys [75]. Importantly, even though CRF receptors have been identified on GnRH1 neurons in the POA of mice [27], tract-tracing studies have thus far failed to show direct connections between CRF and GnRH1 neurons in many species, including teleost fishes. Therefore, it is possible that CRF influences GnRH1 activity in the POA via indirect mechanisms on glutamatergic or other transmitter systems.

### Stimulation of CRF activity in serotonergic brain regions

One candidate mechanism for regulation of CRF signaling is the monoamine neurotransmitter serotonin. Serotonin is well known for its role in modulating social behavior, aggression and sex change in teleost fishes [76], [77]. In addition, serotonin levels are sensitive to CRF activity, and seem to play a role in modulating anxiety-like behavior in fish [21]. Recent tract tracing studies in mice show a robust connection from the hindbrain raphé nucleus, the main source for serotonin in the brain, to GnRH1 neurons in the POA [78]. As subordinate *A. burtoni* have higher serotonergic activity in both the brainstem (which includes the raphé) and the hypothalamus when compared to dominant individuals [79], it is possible that serotonergic signaling serves as an indirect mechanism influencing CRF-mediated GnRH1 activity.

In the rat hypothalamus, immunohistochemical studies show that serotonin-containing fibers overlap with CRF-immunoreactive neurons [80], [81]. There is also evidence that CRF, acting through its two distinct receptors CRFR_1_ and CRFR_2_ in the raphé, can regulate serotonin activity in a topographically distinct pattern [45], [82]. In fishes, social rank and agonistic behavior influence serotonergic activity [83], [84] and consistently, subordinate individuals express elevated levels of serotonin throughout the brain [79], [85], such that serotonin and aggressive behavior are generally inversely correlated. Thus, our results that show increased *CRFR_1_* mRNA in the raphé at 15 minutes after perception of a social opportunity, while *CRF* and *CRFR_1_* mRNA decreased in all other brain regions measured, suggests the raphé CRF activity may be modulating serotonergic release to both stimulate behavioral changes as well as GnRH1 activity. Thus, HPG axis activation during social ascent may be influenced directly through central CRF signaling, via HPA/I axis activity, indirectly through CRF mediation of serotonergic pathways, or some combination of these mechanisms.

## Conclusions

Social transitions in *A. burtoni* occur rapidly in natural colonies, and ascending fish show changes in behavior and body coloration within minutes. Here we show that in subordinate males ascending to a dominant role, *CRF* (Pit, raphé) and *CRFR_1_* (Vc/Vl, POA, Pit) mRNA is down regulated within 15 minutes. We hypothesize that rapidly decreasing CRF activity may disinhibit the HPG axis during a rise in social rank. Our data imply that a reduction/removal of the inhibitory central CRF system may allow for the physiological changes, especially in POA GnRH1 cells, that these ascending fish need to rapidly achieve reproductive viability. It is also possible that CRF activity in the raphé is involved in mediating these responses, as serotonin is closely linked to behavioral and reproductive activity in vertebrates. In species where social rank ordering is a primary mechanism for determining reproductive opportunities, integrated and reciprocal connections between the CRF and serotonergic systems that influence the stress response and those that modulate reproductive capacity would provide a rapid mechanism for prompt changes. These directed changes could support new behaviors and physiology that allow individuals to take advantage of limited social opportunities. The CRF-GnRH1 model proposed here highlights one such possible neurobiological substrate for this type of directed action.

## References

[1] Bale TL, Vale WW (2004) CRF and CRF receptors: role in stress responsivity and other behaviors. Annu Rev Pharmacol Toxicol 44: : 525–557. Available: http://www.ncbi.nlm.nih.gov/pubmed/14744257. Accessed 30 October 2013. 10.1146/annurev.pharmtox.44.101802.121410 14744257

[2] Carrasco GA, Kar V De (2003) L.d (2003) Neuroendocrine pharmacology of stress. Eur J Pharmacol 463: 235–272. 12600714 10.1016/s0014-2999(03)01285-8

[3] Doyon C, Gilmour KM, Trudeau VL, Moon TW (2003) Corticotropin-releasing factor and neuropeptide Y mRNA levels are elevated in the preoptic area of socially subordinate rainbow trout. Gen Comp Endocrinol 133: 260–271 10.1016/S0016-6480(03)00195-3 12928015

[4] Sapolsky RM (1989) Hypercortisolism among socially subordinate wild baboons originates at the CNS level. Arch Gen Psychiatry 46: 1047–1051 10.1001/archpsyc.1989.01810110089012 2554841

[5] Fernald RD, Maruska KP (2012) Social information changes the brain. Proc Natl Acad Sci U S A 109 Suppl: 17194–17199. Available: http://www.ncbi.nlm.nih.gov/pubmed/23045669. 23045669 10.1073/pnas.1202552109 PMC3477382

[6] Desjardins JK, Hofmann H a, Fernald RD (2012) Social context influences aggressive and courtship behavior in a cichlid fish. PLoS One 7: : e32781. Available: http://www.pubmedcentral.nih.gov/articlerender.fcgi?artid=3395714&tool=pmcentrez&rendertype=abstract. Accessed 30 October 2013. 10.1371/journal.pone.0032781 PMC3395714 22807996

[7] Hofmann HA, Benson ME, Fernald RD (1999) Social status regulates growth rate: consequences for life-history strategies. Proc Natl Acad Sci U S A 96: 14171–14176. 10570217 10.1073/pnas.96.24.14171 PMC24209

[8] Maruska KP, Fernald RD (2013) Social Regulation of Male Reproductive Plasticity in an African Cichlid Fish. Integr Comp Biol: 1–13. Available: http://www.ncbi.nlm.nih.gov/pubmed/23613320. 10.1093/icb/ict017 PMC3836007 23613320

[9] Burmeister SS, Jarvis ED, Fernald RD (2005) Rapid behavioral and genomic responses to social opportunity. PLoS Biol 3: e363 10.1371/journal.pbio.0030363 16216088 PMC1255743

[10] Burmeister SS, Kailasanath V, Fernald RD (2007) Social dominance regulates androgen and estrogen receptor gene expression. Horm Behav 51: : 164–170. Available: http://www.ncbi.nlm.nih.gov/pubmed/17081541. Accessed 30 October 2013. 10.1016/j.yhbeh.2006.09.008 PMC1851932 17081541

[11] Maruska KP, Fernald RD (2010) Behavioral and physiological plasticity: rapid changes during social ascent in an African cichlid fish. Horm Behav 58: 230–240 10.1016/j.yhbeh.2010.03.011 20303357 PMC2922674

[12] Maruska KP, Fernald RD (2011) Plasticity of the reproductive axis caused by social status change in an african cichlid fish: II. testicular gene expression and spermatogenesis. Endocrinology 152: 291–302 10.1210/en.2010-0876 21084443 PMC3219049

[13] Maruska KP, Zhang a, Neboori a, Fernald RD (2013) Social opportunity causes rapid transcriptional changes in the social behaviour network of the brain in an African cichlid fish. J Neuroendocrinol 25: 145–157 Available: http://www.ncbi.nlm.nih.gov/pubmed/22958303. 22958303 10.1111/j.1365-2826.2012.02382.x PMC3537875

[14] Williams CL, Nishihara M, Thalabard JC, Grosser PM, Hotchkiss J, et al (1990) Corticotropin-releasing factor and gonadotropin-releasing hormone pulse generator activity in the rhesus monkey. Electrophysiological studies. Neuroendocrinology 52: 133–137. 2125701 10.1159/000125563

[15] Nikolarakis KE, Almeida OF, Herz A (1986) Corticotropin-releasing factor (CRF) inhibits gonadotropin-releasing hormone (GnRH) release from superfused rat hypothalami in vitro. Brain Res 377: 388–390 10.1016/0006-8993(86)90887-5 3524753

[16] Breen KM, Davis TL, Doro LC, Nett TM, Oakley AE, et al (2008) Insight into the neuroendocrine site and cellular mechanism by which cortisol suppresses pituitary responsiveness to gonadotropin-releasing hormone. Endocrinology 149: 767–773 10.1210/en.2007-0773 17962347 PMC2219297

[17] Rivier C, Brownstein M, Spiess J, Rivier J, Valef W (1982) In Vivo Corticotropin-Releasing Factor-Induced Secretion. 110: 272–278. 10.1210/endo-110-1-272 6274623

[18] Lowry C a, Moore FL (2006) Regulation of behavioral responses by corticotropin-releasing factor. Gen Comp Endocrinol 146: 19–27 Available: http://www.ncbi.nlm.nih.gov/pubmed/16426606. 16426606 10.1016/j.ygcen.2005.12.006

[19] Summers CH, Korzan WJ, Lukkes JL, Øverli Ø, Höglund E, et al. (2005) Does serotonin influence aggression? Physiol Biochem Zool 78: : 679–694. Available: http://www.journals.uchicago.edu/doi/abs/10.1086/432139\n http://www.journals.uchicago.edu/doi/abs/10.1086/432139?url_ver=Z39.88-2003&rfr_id=ori:rid:crossref.org&rfr_dat=cr_pub%3dncbi.nlm.nih.gov. 10.1086/432139 16059845

[20] Hoffman CS, Westin TM, Miner HM, Johnson PL, Summers CH, et al (2002) GABAergic drugs alter hypothalamic serotonin release and lordosis in estrogen-primed rats. Brain Res 946: 96–103 Available: http://www.ncbi.nlm.nih.gov/pubmed/12133599. 12133599 10.1016/s0006-8993(02)02867-6

[21] Carpenter RE, Watt MJ, Forster GL, Øverli Ø, Bockholt C, et al (2007) Corticotropin releasing factor induces anxiogenic locomotion in trout and alters serotonergic and dopaminergic activity. Horm Behav 52: 600–611 10.1016/j.yhbeh.2007.07.012 17826776 PMC3889481

[22] Grone BP, Carpenter RE, Maruska KP, Fernald RD, Lee M (2012) Food deprivation explains effects of mouthbrooding on ovaries and steroid hormones, but not brain neuropeptide and receptor mRNAs, in an African cichlid fish. Horm Behav 62: 18–26 10.1016/j.yhbeh.2012.04.012 22561338 PMC3379815

[23] Clements S (2003) Evidence that acute serotonergic activation potentiates the locomotor-stimulating effects of corticotropin-releasing hormone in juvenile chinook salmon (Oncorhynchus tshawytscha). Horm Behav 43: 214–221 Available: http://linkinghub.elsevier.com/retrieve/pii/S0018506X02000272. 12614652 10.1016/s0018-506x(02)00027-2

[24] Sirinathsinghji DJ (1985) Modulation of lordosis behaviour in the female rat by corticotropin releasing factor, beta-endorphin and gonadotropin releasing hormone in the mesencephalic central gray. Brain Res 336: 45–55 Available: http://www.ncbi.nlm.nih.gov/pubmed/2860950. 2860950 10.1016/0006-8993(85)90414-7

[25] Sirinathsinghji DJ (1986) Regulation of lordosis behaviour in the female rat by corticotropin-releasing factor, beta-endorphin/corticotropin and luteinizing hormone-releasing hormone neuronal systems in the medial preoptic area. Brain Res 375: 49–56 10.1016/0006-8993(86)90957-1 3013371

[26] MacLusky NJ, Naftolin F, Leranth C (1988) Immunocytochemical evidence for direct synaptic connections between corticotrophin-releasing factor (CRF) and gonadotrophin-releasing hormone (GnRH)-containing neurons in the preoptic area of the rat. Brain Res 439: 391–395. 3282601 10.1016/0006-8993(88)91501-6

[27] Jasoni CL, Todman MG, Han S-K, Herbison AE (2005) Expression of mRNAs encoding receptors that mediate stress signals in gonadotropin-releasing hormone neurons of the mouse. Neuroendocrinology 82: 320–328 10.1159/000093155 16721036

[28] Fernald RD, Hirata NR (1977) Field Study of Haplochromis-Burtoni Quantitative Behavioral Observations. Anim Behav 25: : 964–975. Available: <Go to ISI>://BIOSIS:PREV197865068493.

[29] Maruska KP, Fernald RD (2010) Reproductive status regulates expression of sex steroid and GnRH receptors in the olfactory bulb. Behav Brain Res 213: 208–217 Available: 10.1016/j.bbr.2010.04.058. 20466023 PMC2902620

[30] Fernald RD, Hirata NR (1977) Field study of Haplochromis burtoni: habitats and co-habitant. Environ Biol Fishes 2: 299–308 10.1007/BF00005997

[31] Davis MR, Fernald RD (1990) Social control of neuronal soma size. J Neurobiol 21: 1180–1188 10.1002/neu.480210804 2273399

[32] Maruska KP, Fernald RD (2010) Steroid receptor expression in the fish inner ear varies with sex, social status, and reproductive state. BMC Neurosci 11: 58 10.1186/1471-2202-11-58 20433748 PMC2876163

[33] Burmeister SS, Munshi RG, Fernald RD (2009) Cytoarchitecture of a cichlid fish telencephalon. Brain Behav Evol 74: 110–120 10.1159/000235613 19729898 PMC2924238

[34] Fernald RD, Shelton LC (1985) The organization of the diencephalon and the pretectum in the cichlid fish, Haplochromis burtoni. J Comp Neurol 238: 202–217 10.1002/cne.902380207 4044911

[35] Munchrath L a, Hofmann H a (2010) Distribution of sex steroid hormone receptors in the brain of an African cichlid fish, Astatotilapia burtoni. J Comp Neurol 518: : 3302–3326. Available: http://www.ncbi.nlm.nih.gov/pubmed/20575061. Accessed 31 October 2013. 10.1002/cne.22401 20575061

[36] Maler L, Sas E, Johnston S, Ellis W (1991) An atlas of the brain of the electric fish Apteronotus leptorhynchus. J Chem Neuroanat 4: 1–38 Available: http://www.ncbi.nlm.nih.gov/pubmed/2012682. 2012682 10.1016/0891-0618(91)90030-g

[37] Cerdá-Reverter JM, Zanuy S, Muñoz-Cueto J a (2001) Cytoarchitectonic study of the brain of a perciform species, the sea bass (Dicentrarchus labrax). I. The telencephalon. J Morphol 247: 217–228 Available: http://www.ncbi.nlm.nih.gov/pubmed/11223929. 11223929 10.1002/1097-4687(200103)247:3<217::AID-JMOR1013>3.0.CO;2-U

[38] Rupp B, Wullimann MF, Reichert H (1996) The zebrafish brain: a neuroanatomical comparison with the goldfish. Anat Embryol (Berl) 194: 187–203 10.1007/BF00195012 8827327

[39] Pepels PPLM, Balm PHM (2004) Ontogeny of corticotropin-releasing factor and of hypothalamic-pituitary-interrenal axis responsiveness to stress in tilapia (Oreochromis mossambicus; Teleostei). Gen Comp Endocrinol 139: 251–265 10.1242/jeb.01316 15560872

[40] Yamamoto N, Parhar IS, Sawai N, Oka Y, Ito H (1998) Preoptic gonadotropin-releasing hormone (GnRH) neurons innervate the pituitary in teleosts. Neurosci Res 31: 31–38 10.1016/S0168-0102(98)00022-4 9704976

[41] Bushnik TL, Fernald RD (1995) The population of GnRH-containing neurons showing socially mediated size changes project to the pituitary in a teleost, Haplochromis burtoni. Brain Behav Evol 46: 371–377 10.1159/000113287 8719758

[42] Hornung J-P (2003) The human raphe nuclei and the serotonergic system. J Chem Neuroanat 26: 331–343 10.1016/j.jchemneu.2003.10.002 14729135

[43] Cooper MA, Grober MS, Nicholas CR, Huhman KL (2009) Aggressive encounters alter the activation of serotonergic neurons and the expression of 5-HT1A mRNA in the hamster dorsal raphe nucleus. Neuroscience 161: 680–690 10.1016/j.neuroscience.2009.03.084 19362123 PMC2692818

[44] Cooper MA, Huhman KL (2007) Corticotropin-releasing factor receptors in the dorsal raphe nucleus modulate social behavior in Syrian hamsters. Psychopharmacology (Berl) 194: 297–307 10.1007/s00213-007-0849-1 17581742 PMC2714987

[45] Lowry CA, Rodda JE, Lightman SL, Ingram CD (2000) Corticotropin-releasing factor increases in vitro firing rates of serotonergic neurons in the rat dorsal raphe nucleus: evidence for activation of a topographically organized mesolimbocortical serotonergic system. J Neurosci 20: 7728–7736 doi:20/20/7728 [pii] 11027235 10.1523/JNEUROSCI.20-20-07728.2000 PMC6772886

[46] Waselus M, Nazzaro C, Valentino RJ, Van Bockstaele EJ (2009) Stress-induced redistribution of corticotropin-releasing factor receptor subtypes in the dorsal raphe nucleus. Biol Psychiatry 66: 76–83 10.1016/j.biopsych.2009.02.014 19362706 PMC2728006

[47] Majzoub JA (2006) Corticotropin-releasing hormone physiology. Eur J Endocrinol 155: S71–S76 10.1530/eje.1.02247

[48] Chen C-C, Fernald RD (2008) Sequences, expression patterns and regulation of the corticotropin-releasing factor system in a teleost. Gen Comp Endocrinol 157: 148–155 10.1016/j.ygcen.2008.04.003 18501902 PMC3357958

[49] Zhao S, Fernald RD (2005) Comprehensive algorithm for quantitative real-time polymerase chain reaction. J Comput Biol 12: 1047–1064 Available: http://www.ncbi.nlm.nih.gov/pubmed/16241897. 16241897 10.1089/cmb.2005.12.1047 PMC2716216

[50] Maruska KP, Fernald RD (2011) Social regulation of gene expression in the hypothalamic-pituitary-gonadal axis. Physiol 26: 412–423 Available: http://www.ncbi.nlm.nih.gov/pubmed/22170959. 10.1152/physiol.00032.2011 22170959

[51] Au TM, Greenwood AK, Fernald RD (2006) Differential social regulation of two pituitary gonadotropin-releasing hormone receptors. Behav Brain Res 170: 342–346 10.1016/j.bbr.2006.02.027 16580741

[52] Zhao S, Kelm RJ, Fernald RD (2010) Regulation of gonadotropin-releasing hormone-1 gene transcription by members of the purine-rich element-binding protein family. Am J Physiol Endocrinol Metab 298: E524–E533 10.1152/ajpendo.00597.2009 19996387 PMC2838525

[53] Bustin SA, Benes V, Nolan T, Pfaffl MW (2005) Quantitative real-time RT-PCR - a perspective. J Mol Endocrinol 34: 597–601 Available: http://jme.endocrinology-journals.org/cgi/content/abstract/34/3/597. 15956331 10.1677/jme.1.01755

[54] Mitter K, Kotoulas G, Magoulas A, Mulero V, Sepulcre P, et al (2009) Evaluation of candidate reference genes for QPCR during ontogenesis and of immune-relevant tissues of European seabass (Dicentrarchus labrax). Comp Biochem Physiol B Biochem Mol Biol 153: 340–347 10.1016/j.cbpb.2009.07.008 19398033

[55] Rodaros D, Caruana DA, Amir S, Stewart J (2007) Corticotropin-releasing factor projections from limbic forebrain and paraventricular nucleus of the hypothalamus to the region of the ventral tegmental area. Neuroscience 150: 8–13 10.1016/j.neuroscience.2007.09.043 17961928

[56] Rivier C, Rivest S (1991) Effect of stress on the activity of the hypothalamic-pituitary-gonadal axis: peripheral and central mechanisms. Biol Reprod 45: 523–532 10.1095/biolreprod45.4.523 1661182

[57] Ferin M (2006) Stress and the Reproductive System. Stress Int J Biol Stress 62: 2627–2696 Available: http://www.sciencedirect.com/science/article/B84D6-4NGKRPJ-1F/2/026393f57d6f225d16c82f92b1988107.

[58] Dobson H, Smith RF (2000) What is stress, and how does it affect reproduction? Anim Reprod Sci 60–61: 743–752 10.1016/S0378-4320(00)00080-4 10844239

[59] Suh BY, Liu JH, Berga SL, Quigley ME, Laughlin GA, et al (1988) Hypercortisolism in patients with functional hypothalamic-amenorrhea. J Clin Endocrinol Metab 66: 733–739 10.1097/00006254-198811000-00016 3346352

[60] Suter DE, Schwartz NB, Ringstrom SJ (1988) Dual role of glucocorticoids in regulation of pituitary content and secretion of gonadotropins. Am J Physiol 254: E595–E600. 3129945 10.1152/ajpendo.1988.254.5.E595

[61] Fox HE, White SA, Kao MH, Fernald RD (1997) Stress and dominance in a social fish. J Neurosci 17: 6463–6469. 9236253 10.1523/JNEUROSCI.17-16-06463.1997 PMC6568347

[62] Sawchenko PE, Swanson LW (1985) Localization, colocalization, and plasticity of corticotropin-releasing factor immunoreactivity in rat brain. Fed Proc 44: 221–227. 2981743

[63] Doyon C, Trudeau VL, Moon TW (2005) Stress elevates corticotropin-releasing factor (CRF) and CRF-binding protein mRNA levels in rainbow trout (Oncorhynchus mykiss). J Endocrinol 186: 123–130 Available: http://www.ncbi.nlm.nih.gov/pubmed/16002542. 16002542 10.1677/joe.1.06142

[64] Dudas B, Merchenthaler I (2006) Three-dimensional representation of the neurotransmitter systems of the human hypothalamus: inputs of the gonadotrophin hormone-releasing hormone neuronal system. J Neuroendocrinol 18: 79–95 10.1111/j.1365-2826.2005.01398.x 16420277

[65] Rivest S, Plotsky PM, Rivier C (1993) CRF alters the infundibular LHRH secretory system from the medial preoptic area of female rats: possible involvement of opioid receptors. Neuroendocrinology 57: 236–246 10.1159/000126365 8389996

[66] Li XF, Lin YS, Kinsey-Jones JS, Milligan SR, Lightman SL, et al (2011) The role of the bed nucleus of the stria terminalis in stress-induced inhibition of pulsatile luteinising hormone secretion in the female rat. J Neuroendocrinol 23: 3–11 10.1111/j.1365-2826.2010.02071.x 21073554

[67] Sisk CL, Foster DL (2004) The neural basis of puberty and adolescence. Nat Neurosci 7: 1040–1047 10.1038/nn1326 15452575

[68] Ebling FJP (2005) The neuroendocrine timing of puberty. Reproduction 129: 675–683 10.1530/rep.1.00367 15923383

[69] Parent A-S, Matagne V, Bourguignon J-P (2005) Control of puberty by excitatory amino acid neurotransmitters and its clinical implications. Endocrine 28: 281–286 10.1385/ENDO:28:3:281 16388117

[70] Van den Pol AN (1993) Glutamate and GABA presence and action in the suprachiasmatic nucleus. J Biol Rhythms 8 Suppl: S11–S15. 7903876

[71] Goldsmith PC, Thind KK, Perera AD, Plant TM (1994) Glutamate-immunoreactive neurons and their gonadotropin-releasing hormone-neuronal interactions in the monkey hypothalamus. Endocrinology 134: 858–868. 7905410 10.1210/endo.134.2.7905410

[72] Trudeau VL, Sloley BD, Peter RE (1993) Testosterone enhances GABA and taurine but not N-methyl-D,L-aspartate stimulation of gonadotropin secretion in the goldfish: possible sex steroid feedback mechanisms. J Neuroendocrinol 5: 129–136. 8097942 10.1111/j.1365-2826.1993.tb00372.x

[73] Flett P, Kftaak GVANDER, Leatherland JF (1994) Effects of Excitatory Amino Acids on In Vivo and In Vitro Gonadotropin and Growth Hormone Secretion in Testosterone-Primed Immature Rainbow Trout, Oncorhynchus mykiss. 399.

[74] Densmore VS, Urbanski HF (2003) Relative effect of gonadotropin-releasing hormone (GnRH)-I and GnRH-II on gonadotropin release. J Clin Endocrinol Metab 88: 2126–2134 10.1210/jc.2002-021359 12727965

[75] Plant TM, Gay VL, Marshall GR, Arslan M (1989) Puberty in monkeys is triggered by chemical stimulation of the hypothalamus. Proc Natl Acad Sci U S A 86: 2506–2510 10.1073/pnas.86.7.2506 2648405 PMC286942

[76] Clotfelter ED, McNitt MM, Carpenter RE, Summers CH (2010) Modulation of monoamine neurotransmitters in fighting fish Betta splendens exposed to waterborne phytoestrogens. Fish Physiol Biochem 36: 933–943 10.1007/s10695-009-9370-2 20012186

[77] Lorenzi V, Carpenter RE, Summers CH, Earley RL, Grober MS (2009) Serotonin, social status and sex change in the bluebanded goby Lythrypnus dalli. Physiol Behav 97: 476–483 10.1016/j.physbeh.2009.03.026 19345236 PMC2683889

[78] Campbell RE, Herbison AE (2007) Definition of brainstem afferents to gonadotropin-releasing hormone neurons in the mouse using conditional viral tract tracing. Endocrinology 148: 5884–5890 10.1210/en.2007-0854 17823269 PMC6101187

[79] Winberg S, Winberg Y, Fernald RD (1997) Effect of social rank on brain monoaminergic activity in a cichlid fish. Brain Behav Evol 49: 230–236 10.1159/000112994 9096910

[80] Liposits Z, Phelix C, Paull WK (1987) Synaptic interaction of serotonergic axons and corticotropin releasing factor (CRF) synthesizing neurons in the hypothalamic paraventricular nucleus of the rat. Histochemistry 86: 541–549 10.1007/BF00489545 3497137

[81] Sawchenko PE, Swanson LW, Steinbusch HW, Verhofstad AA (1983) The distribution and cells of origin of serotonergic inputs to the paraventricular and supraoptic nuclei of the rat. Brain Res 277: 355–360 10.1016/0006-8993(83)90945-9 6357352

[82] Staub DR, Evans AK, Lowry CA (2006) Evidence supporting a role for corticotropin-releasing factor type 2 (CRF2) receptors in the regulation of subpopulations of serotonergic neurons. Brain Res 1070: 77–89 10.1016/j.brainres.2005.10.096 16403469

[83] Nilsson GE, Winberg S (1993) Roles of brain monoamine neurotransmitters in agonistic behaviour and stress reactions, with particular reference to fish. Comp Biochem Physiol Part C Pharmacol Toxicol Endocrinol 106: 597–614 10.1016/0742-8413(93)90216-8

[84] Teles MC, Dahlbom SJ, Winberg S, Oliveira RF (2013) Social modulation of brain monoamine levels in zebrafish. Behav Brain Res 253: 17–24 Available: http://www.ncbi.nlm.nih.gov/pubmed/23850359. 23850359 10.1016/j.bbr.2013.07.012

[85] Dahlbom SJ, Backström T, Lundstedt-Enkel K, Winberg S (2012) Aggression and monoamines: Effects of sex and social rank in zebrafish (Danio rerio). Behav Brain Res 228: 333–338 10.1016/j.bbr.2011.12.011 22192379

